# Understanding the Impact of Uterine Fibroids on Human Endometrium Function

**DOI:** 10.3389/fcell.2021.633180

**Published:** 2021-05-25

**Authors:** Antonia Navarro, Maria Victoria Bariani, Qiwei Yang, Ayman Al-Hendy

**Affiliations:** Department of Obstetrics and Gynecology, University of Chicago, Chicago, IL, United States

**Keywords:** uterine fibroids, endometrium, heavy menstrual bleeding, endometrial receptivity, implantation, subfertility, transforming growth factor beta

## Abstract

Uterine fibroids (leiomyomas) are the most common benign gynecological tumors in women of reproductive age worldwide. They cause heavy menstrual bleeding, usually leading to severe anemia, pelvic pain/pressure, infertility, and other debilitating morbidities. Fibroids are believed to be monoclonal tumors arising from the myometrium, and recent studies have demonstrated that fibroids actively influence the endometrium globally. Studies suggest a direct relationship between the number of fibroids removed and fertility problems. In this review, our objective was to provide a complete overview of the origin of uterine fibroids and the molecular pathways and processes implicated in their development and growth, which can directly affect the function of a healthy endometrium. One of the most common characteristics of fibroids is the excessive production of extracellular matrix (ECM) components, which contributes to the stiffness and expansion of fibroids. ECM may serve as a reservoir of profibrotic growth factors such as the transforming growth factor β (TGF-β) and a modulator of their availability and actions. Fibroids also elicit mechanotransduction changes that result in decreased uterine wall contractility and increased myometrium rigidity, which affect normal biological uterine functions such as menstrual bleeding, receptivity, and implantation. Changes in the microRNA (miRNA) expression in fibroids and myometrial cells appear to modulate the TGF-β pathways and the expression of regulators of ECM production. Taken together, these findings demonstrate an interaction among the ECM components, TGF-β family signaling, miRNAs, and the endometrial vascular system. Targeting these components will be fundamental to developing novel pharmacotherapies that not only treat uterine fibroids but also restore normal endometrial function.

## Introduction

Uterine fibroids (UFs), also known as leiomyomas, are benign tumors in women of reproductive age. Despite their benign nature, they are able to undergo rapid and significant growth (Andersen and Barbieri, 1995). They cause irregular and heavy menstrual bleeding (HMB), leading to severe anemia, dysmenorrhea, pelvic pressure and pain, urinary incontinence, dyspareunia, infertility, preterm labor, and early and recurrent pregnancy loss (Stewart, 2001; Wallach and Vlahos, 2004). UFs are present in more than 70% of women, become symptomatic in approximately 30% of women, and are the most common clinical indication for hysterectomy that prematurely ends a woman’s reproductive life (Walker and Stewart, 2005). African American women are more likely to develop UFs at an early age and to present more severe clinical symptoms compared to Caucasian women (Kjerulff et al., 1996). Other risk factors for UFs include age, obesity, hypovitaminosis D, and endogenous and exogenous hormonal factors (Pavone et al., 2018; Ali et al., 2019; Bariani et al., 2020). Despite the high prevalence of UFs, there are no approved effective pharmacotherapies, and surgery remains the main option for UF treatment (Bulun, 2013). UF prevalence is a personal and economic burden, within an estimated healthcare cost of United States $34 billion annually in the United States (Cardozo et al., 2012).

UFs cause HMB and poor uterine receptivity and implantation leading to infertility, two major female reproductive disorders affecting millions of women in the United States and globally. Both disorders reflect endometrial dysfunctions caused by the presence of UFs, and the degree of dysfunction appears associated with the location and size of the UFs. There is a significant gap in our understanding of how UFs affect endometrial function. It is generally agreed that the expansion in the endometrial surface area caused by UFs leads to greater menstrual bleeding and transformations in the shape of uterine cells that affect gene expression and function. Yet, recent studies have demonstrated that UFs actively influence not only the adjacent endometrium but also the uterus as a whole (Rackow and Taylor, 2010). We conducted a literature review to highlight new and significant insights into endometrial and UF biology, with the goal of elucidating the effects of UFs on human endometrial function, particularly HMB, infertility, and pregnancy complications.

## Methods

We used several strategies to identify primary research literature, review articles, and book chapters related to UFs and the negative impacts of UFs on endometrial function, focusing on HMB, infertility, and pregnancy complications. We did not place restrictions on year of publication and included all relevant publications up to November 2020. We performed PubMed and Google Scholar searches to identify relevant articles using the following keywords either alone or in combination with “uterine fibroid(s), uterine leiomyoma, and endometrium,” “heavy menstrual bleeding,” “recurrent pregnancy loss,” “miscarriage,” “early pregnancy loss,” “infertility,” and “subfertility.” We also considered additional pertinent articles included as references in the downloaded articles. With this review, we summarize and expand on what is presently known regarding the influence of UFs on the endometrium and the associated clinical consequences of UFs such as HMB and infertility.

## Results

### Origin of Uterine Fibroids

UFs are monoclonal tumors (Holdsworth-Carson et al., 2014), and increasing evidence indicates that they arise from a single myometrial stem cell (MMSC) (Mas et al., 2012; Yin et al., 2015). MMSCs constitute a small proportion of the total population of cells and express specific surface markers that distinguish them from the bulk of other cells (Mas et al., 2015). The plasticity of MMSCs during development and tissue maintenance permits the acquisition of mutations or aberrant cellular reprogramming *via* epigenetic mechanisms. Consequently, normal MMSCs can be converted into tumor-initiating stem cells (TICs) that are able to initiate UF development. The most common genetic drivers associated with the development of UFs are somatic mutations present in exons 1 and 2 of the *MED12* gene, which encodes a subunit of the mediator complex, a co-activator involved in the transcription of nearly all RNA polymerase II-dependent genes (Makinen et al., 2011). In addition to *MED12* mutations, which account for ∼70% of UFs (Je et al., 2012; Kämpjärvi et al., 2014), a proportionally smaller fraction of UFs is thought to arise from genetic alterations leading to the overexpression of high-mobility group AT-hook 2 (HMGA2, ∼20%), biallelic inactivation of fumarate hydratase (FH, ∼2%) (Mehine et al., 2016), and disruption of the COL4A6 locus (∼3%).

### Factors Implicated in Uterine Fibroid Development and Growth and Their Influence on Endometrial Biology

**FIGURE 1:**
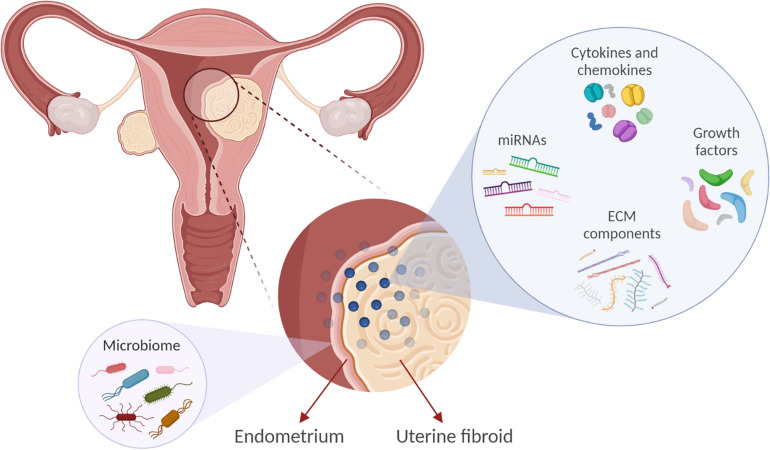
Factors implicated in uterine fibroid (UF) development and growth may influence the endometrial biology. Extracellular matrix (ECM) components, microRNAs (miRNAs), growth factors, cytokines, and chemokines are involved in UF development. These factors may also affect endometrial cell growth and function and vessel remodeling, thereby contributing to the increased incidence of reproductive complications such as heavy and irregular menses and infertility. In addition, we propose that UFs may impact the endometrial microbiome composition.

The exact cellular and molecular mechanisms that direct and control the development and growth of UFs are not clearly elucidated. However, several factors have been implicated in the development and growth of UFs, such as cytokines, chemokines, growth factors, extracellular matrix (ECM) components, factors involved in the DNA damage response and inflammation, vasoactive substances, and microRNAs (Figure 1). The factors expressed and secreted by UFs could affect endometrial cell growth and function and vessel remodeling, thereby contributing to the increased incidence of HMB, irregular menses, and infertility (Table 1). One of the main characteristics of UFs is a remarkably excessive production of ECM components including collagens, fibronectin, proteoglycans, and laminins (Norian et al., 2009).

**TABLE 1:** Differential expressions of the factors involved in uterine fibroid (UF) development and growth and its effect on the endometrium.

**Factor**	**Expression in UF**	**Effect on endometrium**
**Cytokines**		
TNF-α	• Increased expression in UFs compared with the adjacent myometrium (Kurachi et al., 2001) • Elevated serum levels in women with clinically symptomatic UFs (Ciebiera et al., 2018a)	• Involved in menstrual shedding process and bleeding (Tabibzadeh, 1996) • Increased concentrations in menses blood flow in women with HMB (Malik et al., 2006)
GM-CSF	• UF smooth muscle cells isolated from patients express higher GM-CSF mRNA and protein levels than myometrial smooth muscle cells (Chegini et al., 1999)	• GM-CSF improves endometrial regeneration (Liu et al., 2020) and increase endometrial thickness (Mao et al., 2020)
IL-33	• Serum levels of IL-33 are significantly higher in UFs patients as compared to the controls (Santulli et al., 2013)	• Modulates the timely recruitment of neutrophils and lymphocytes into the endometrium (Arici et al., 1998a) • Critical regulator of inflammation and vascularization (Miller et al., 2017)
**Chemokines**		
IL-8	• Expression of IL-8 and its receptor type A is weaker in UFs compared with adjacent myometrium (Senturk et al., 2001)	• May act as an autocrine growth factor in the endometrium (Arici et al., 1998b; Arici, 2002)
MCP-1	• mRNA levels in UF samples are higher than in the myometrium samples (Sozen et al., 1998)	• Control macrophage endometrial migration (Arici et al., 1999)
**Growth factors**		
TGF-β	• Upregulated in UFs compared with adjacent myometrium (Hoffman et al., 2004; Norian et al., 2009; Ciebiera et al., 2017)	• Involved in proliferation and remodeling during the menstrual cycle (Omwandho et al., 2010) • Preparation of the endometrium for implantation (Jones et al., 2006)
VEGF	• Higher expression in UFs compared to myometrium (Gentry et al., 2001; Sanci et al., 2011; Tal and Segars, 2014)	Key factor in endometrial angiogenesis during menstrual cycles and early pregnancy (Sugino et al., 2002) • Altered expression is associated with HMB (Smith, 1998)
PDGF	• Increased expression of PDGF-C in UFs compared with the adjacent myometrium (Hoffman et al., 2004; Hwu et al., 2008; Suo et al., 2009)	Stimulates the proliferation of endometrial stromal cells (Matsumoto et al., 2005) • Involved in endometrial regeneration (Wang et al., 2020)
EGF	• Inconsistent results regarding EGF expression in UFs compared to the adjacent myometrium. (higher: Harrison-Woolrych et al., 1994; no difference: Vollenhoven et al., 1995; lower: Dixon et al., 2000)	• Central role in the regulation of cyclical growth and shedding of the endometrium (Ejskjær et al., 2005) • Critical for endometrial function during early pregnancy (Large et al., 2014)
ECM components	• Collagen, fibronectin, and proteoglycans have been shown to be upregulated in UFs compared to the adjacent myometrium (Islam et al., 2018).	• Structure, organization, and molecular composition of the endometrial ECM are modified during events such as menstrual cycle and decidualization (Tanaka et al., 2009; Favaro et al., 2014; Okada et al., 2018).
**MicroRNAs**		
Let-7 family	• Expression upregulated in UFs compared with matched myometrium (Wang et al., 2007)	• Associated with endometrial receptivity (Inyawilert et al., 2015; Liu et al., 2016)
miR-21	• The most highly upregulated miRNA in UFs (Wang et al., 2007) • Increased expression in UFs compared with adjacent myometrium (Marsh et al., 2008)	• Potential influence on endometrial genes associated with cell cycle progression and apoptotic processes (Pan et al., 2007)
miR-29b/c	• UFs expressed significantly lower levels of miR-29b (Wang et al., 2007) and miR-29c compared to the myometrium (Chuang and Khorram, 2016; Marsh et al., 2016).	• miR-29c dysregulation can alter endometrial receptivity, endometrial epithelial adhesive capacity, and implantation (Griffiths et al., 2019).
miR-200c	• Downregulated in UFs compared to myometrial tissue (Chuang et al., 2012b)	• Involved in the hormonal regulation of epithelial cell proliferation in human endometrium by E2 and P4 (Kuokkanen et al., 2010) • Involved in endometrial receptivity (Liu et al., 2016)

*E2, estrogen; *ECM*, extracellular matrix; *EGF*, epidermal growth factor; *GM-CSF*, granulocyte macrophage-colony-stimulating factor; *HMB*, heavy menstrual bleeding; *IL-33*, interleukin 33; *IL-8*, interleukin 8; *MCP-1*, monocyte chemoattractant protein-1; *P4*, progesterone; *PDGF*, platelet-derived growth factor; *TGF-*β, transforming growth factor beta; *TNF-*α, tumor necrosis factor alpha; *VEGF*, vascular endothelial growth factor.*

### Extracellular Matrix Components and UFs

Extracellular Matrix Component accumulation and remodeling are thought to be critical in the transformation of the myometrium into UFs. Pivotal ECM components including collagen, fibronectin, and proteoglycans are upregulated in UFs compared to the adjacent myometrium (reviewed in Islam et al., 2018) and may be responsible for the increased tissue stiffness and decreased stretch. Interestingly, cells can sense and respond to mechanical stimuli from the environment, such as stretch or compression, by converting them into biochemical signals (Leppert et al., 2014). Furthermore, the ECM may serve as a reservoir of profibrotic growth factors such as the transforming growth factor-β (TGF-β), thus acting as a modulator of their availability and actions (Islam et al., 2018). Beyond its structural role, the ECM is involved in various cellular processes, including cell proliferation and cell death. The structure, organization, and molecular composition of the endometrial ECM are significantly modified during the menstrual cycle and decidualization (Tanaka et al., 2009; Favaro et al., 2014; Okada et al., 2018). In this respect, changes in the ECM environment due to the presence of UFs can significantly disturb normal endometrial physiological functions. UFs may elicit mechanotransduction changes that result in a decreased uterine wall contractility and an increased myometrial rigidity, which in turn affect normal biological uterine functions like menstrual bleeding, receptivity, and implantation. Depending on the location and size of the UFs, an increase in stiffness can affect the endometrium locally by significantly altering stretch and stress and affect gene expression globally (Rogers et al., 2008; Norian et al., 2012). The physical presence of UFs also affects the function of the endometrium, for example by obstructing the transport of gametes or embryo (Deligdish and Loewenthal, 1970) and hindering implantation by altering the normal patterns of myometrial contractions (Lyons et al., 1991). UFs also impair endometrial decidualization in the mid-luteal window of implantation by altering the endomyometrial junctional (EMJ) zone and significantly reducing the concentrations of both macrophages and uterine natural killer (uNK) cells (Kitaya and Yasuo, 2010b) and by altering steroid receptors (Brosens et al., 2003; Tocci et al., 2008). Another important contributor to conception and implantation is uterine peristalsis; the physical presence of UFs causes a decrease in the acceleration of myometrial peristalsis in the mid-luteal period (Kido et al., 2014). Conditions related to uterine peristalsis may contribute to the pathogenesis of several disorders and may impair sperm and embryo transport as well as implantation (Yoshino et al., 2010).

### MicroRNAs in the Regulation of Uterine Fibroids and Their Effects on the Endometrium

MicroRNAs (miRNAs) are small non-coding RNA molecules of approximately 22 nucleotides that function as posttranscriptional regulators of gene expression, affecting a wide array of physiological and pathological processes. miRNAs not only act inside cells but are also released by cells into the extracellular environment to act as autocrine, paracrine, and/or endocrine modulators in recipient cells. Consequently, miRNAs produced and secreted by UFs may influence the entire endometrium. Ali et al. (2020) summarized that multiple studies have reported differential miRNA expressions in UFs compared with matched healthy myometrium. Specifically, UFs have significantly dysregulated the levels of the let-7 family members, miR-21, miR-29b/c, and miR-200c, among others (Wang et al., 2007; Marsh et al., 2008; Chuang et al., 2012a,b). Importantly, the expressions of let-7 miRNAs are significantly upregulated in UFs compared to the matched myometrium, with higher levels of let-7 miRNAs in small UFs (≤3 cm) compared to large UFs (>10 cm) (Wang et al., 2007). Notably, let-7 family members negatively regulated HMGA2 (Wang et al., 2007) and are associated with endometrial receptivity (Inyawilert et al., 2015; Liu et al., 2016). Wang et al. (2007) described miR-21 as the most highly upregulated miRNA in UFs. Interestingly, miR-21 is differentially expressed in endometrial stromal cells and glandular epithelial cells (Nothnick, 2016). Within the miR-29 family, miR-29c expression is downregulated in UFs compared with the myometrium; this miRNA targets the ECM and DNA methylation enzymes (Chuang and Khorram, 2016). Within the endometrium of fertile women, miR-29c is differentially regulated across the fertile menstrual cycle: it is elevated in the mid-secretory, receptive phase compared to the proliferative phase (Kuokkanen et al., 2010). This finding suggests that miR-29c may influence endometrial genes associated with cell cycle progression and apoptotic processes. Furthermore, miR-29c expression is linked to infertility; it is upregulated in the early secretory and mid-secretory phases in the endometrium of infertile women compared to the fertile endometrium in the same phase (Griffiths et al., 2019).

Conversely, miR-200c levels are downregulated in UFs compared to the myometrial tissue, with evidence suggesting a biological role in UF pathophysiology (Chuang et al., 2012b). Moreover, aberrant expression of miR-200c varies by ethnicity, with much lower levels in UF samples from African Americans compared with Caucasian samples (Chuang et al., 2012b). The expression of miR-200c is significantly upregulated in mid-secretory cycle phase samples, and this miRNA is predicted to target many cell cycle genes (Kuokkanen et al., 2010). A very recent study demonstrated that the long non-coding RNA X-inactive specific (XIST) is expressed at higher levels in UFs compared with normal myometrium and that it acts as a molecular sponge for both miR-29c and miR-200c, downregulating the levels of these miRNAs in UFs (Chuang et al., 2020).

It is important to highlight that miRNAs target genes involved in cell growth (miRNA-21/TGF-β), ECM remodeling (miRNA-29/COL1A1 and COL3A), angiogenesis (miRNA-200c, VEGF), and inflammation (miRNA-93/IL-8), leading to complex regulatory networks in UFs that can affect the endometrium (Chuang et al., 2012a,b; Karmon et al., 2014; Ciebiera et al., 2020). Furthermore, steroid hormone signaling, which is crucial in both UF development and endometrial function, regulates miRNAs and *vice versa* (Klinge, 2009; Yuan et al., 2015; Gilam et al., 2017). Consequently, the interaction of multiple active molecules in and around UFs drives the creation of an abnormal endometrial environment leading to adverse menstrual and pregnancy-related outcomes.

### DNA Damage and Repair in Uterine Fibroids

DNA damage can give rise to tumor initiation and progression. Diverse types of DNA damage can be repaired by different mechanisms, such as homologous recombination (HR), non-homologous end joining (NHEJ), and mismatch repair (MMR), among others. Impaired DNA damage repair can provoke genomic instability and lead to genetic alterations. Previous studies from our group revealed the downregulation of several DNA damage repair genes in UFs compared with the adjacent myometrium in women with UFs (Yang et al., 2016; Ali et al., 2019). Prusinski Fernung et al. (2019) compared DNA repair in Stro-1^+^/CD44^+^ MMSCs isolated from human UFs and the adjacent myometrium, revealing increased DNA damage and altered DNA damage repair gene expression and signaling in UFs.

The Eker rat is a unique model to study UF development and the role of early-life exposure to endocrine-disrupting chemicals in UF etiology. We used this model to reveal the accumulation of DNA damage in MMSCs isolated form 5-month-old Eker rats in response to developmental diethylstilbestrol (DES, an endocrine-disrupting chemical) exposure (Prusinski Fernung et al., 2018; Elkafas et al., 2020). In addition, we found that the ability to repair DNA double-strand breaks is impaired in DES-MMSCs compared with vehicle (VEH)-MMSCs.

### Endometrial Dysfunction Caused by Uterine Fibroids: Heavy Menstrual Bleeding and Poor Receptivity and Implantation Leading to Infertility

#### Uterine Fibroids and Heavy Menstrual Bleeding

**FIGURE 2:**
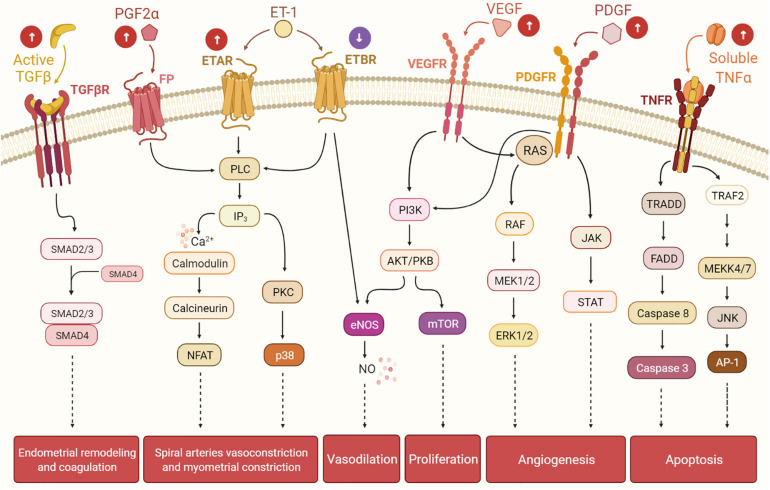
The presence of uterine fibroids (UFs) may interfere with the endometrial pathways involved in the menstrual cycle, leading to heavy menstrual bleeding. Balance among hormones, growth factors, cytokines, and other factors regulates the cyclic endometrial growth and bleeding. Transforming growth factor-beta (TGF-β), its receptors (TGF-βR), and downstream SMADs are important for endometrial remodeling during menses, and excessive levels of these factors may suppress the gene expressions of the fibrinolytic and anticoagulant components. Prostaglandin F2α (PGF2α) and endothelin-1 (ET-1) are involved in spiral artery vasoconstriction and myometrial constriction during the menstruation period. Vascular endothelial growth factor (VEGF), the most specific endothelial cell growth factor, and platelet-derived growth factor (PDGF) play a role in endometrial angiogenesis, an essential process of endometrial renewal. Nitric oxide (NO) is produced downstream of ET-1 and VEGF signaling, and it is a potent vasodilator. Tumor necrosis factor-alpha (TNF-α) contributes to the process of menstrual shedding and bleeding through the induction of apoptosis. *White arrows within circles* indicate uterine changes due to UFs presence, which may dysregulate normal endometrium activity, causing excessive endometrium development and, eventually, heavy menstrual bleeding.

The knowledge gap that links UFs to HMB has limited the development of non-invasive treatment options. HMB is the most common type of abnormal uterine bleeding in women with UFs, and it is commonly accompanied by dysmenorrhea (ACOG Practice Bulletin, 2008; Zimmermann et al., 2012). HMB leads to frequent visits to the emergency room and is the number one indication for hysterectomy (Côté et al., 2003). Women with UF-associated HMB also have a higher risk of developing depression, emotional distress, anxiety, marital strife, and loss of intellectual and work productivity, all of which significantly affect quality of life (Marsh et al., 2014). Menstrual bleeding is a multifaceted combination of interacting processes including angiogenesis, vasodilation, vasoconstriction, coagulation, and inflammation. It is believed that mainly bulky submucosal and intramural UFs affect the normal contractions of the myometrium during menstruation. In normal menstrual cycles, myometrial contractions help to expel uterine menstruation products and reduce loss of blood from endometrial vessels; women with UFs have abnormal myometrial contractions leading to heavy and prolonged menstrual bleeding (Bulun, 2013). The most significant menstrual vasoconstrictors are endothelin-1 (ET1) and prostaglandin F2α (PGFα). ET1 is a strong vasoconstrictor that triggers myometrial contraction and mitogenesis (Masaki, 1993; Maybin and Critchley, 2015). It is mainly expressed in the endometrium, where it is involved in spiral arteriole vasoconstriction and blood flow. ET1 works by binding to its receptors: endothelin type A receptor (ETAR) and endothelin type B receptor (ETBR). Interestingly, women with UFs have greater endometrial expression of ETAR and a lower expression of ETBR compared to normal endometrium. The imbalance in the expressions of ETAR and ETBR in women with UFs may alter ET1 signaling, leading to faulty vasoconstriction, abnormal uterine contractions, and excessive and prolonged menstrual blood flow. There is general consensus that women with UFs and HMB exhibit more dilated endometrial stromal venous spaces compared to women without UFs. Abnormal vasoconstriction might be one of the possible mechanisms underlying HMB (Farrer-Brown et al., 1971). The receptors of the vasoconstrictor PGF2α are expressed in the healthy endometrium and control uterine contractions. Women with UFs have higher levels of endometrial PGF2α, which results in abnormal uterine contractions that could further contribute to HMB (Figure 2; Pekonen et al., 1994; Miura et al., 2006).

Numerous other factors, including cytokines, chemokines, and inflammatory molecules, play important roles in the endometrium during menstrual bleeding and may contribute to UF biology and pathophysiology. Tumor necrosis factor alpha (TNF-α) (Ciebiera et al., 2018b), interleukin 1 (IL-1), IL-11 (Luo et al., 2005), IL-13, IL-15, IL-33 (Santulli et al., 2013), interferon gamma (IFN-γ), and granulocyte–macrophage colony-stimulating factor (G-CSF) (Chegini et al., 1999) are involved in UF pathogenesis. Specifically, TNF-α participates in tissue homeostasis and systemic inflammation, and it is also related to UF-associated HMB (Ciebiera et al., 2018b). TNF-α expression levels are higher in UFs compared with the adjacent myometrium (Kurachi et al., 2001). Moreover, Ciebiera et al. (2018a) demonstrated that TNF-α serum levels are elevated in women with clinically symptomatic UFs. TNF-α is elevated in the menstrual effluent of women with HMB (Malik et al., 2006); thus, this molecule may act as an important local signal that contributes to the process of menstrual shedding and bleeding in UFs (Tabibzadeh, 1996).

Chemokines are a family of chemoattractant cytokines that regulate the infiltration of immune cells subsets, such as leukocytes, into tumors (Nagarsheth et al., 2017) as well as into the endometrium during the normal menstrual cycle (Thiruchelvam et al., 2013). IL-8, which chemoattracts neutrophils, is secreted by several cell types and contributes significantly to various disease-associated processes, including tissue injury, fibrosis, and angiogenesis (Russo et al., 2014). Senturk et al. (2001) demonstrated higher levels of IL-8 and its receptor in the myometrium immediately surrounding the UF compared with the UF itself. Within the endometrium, the IL-8 messenger RNA (mRNA) levels fluctuate throughout the menstrual cycle, with significantly higher expression in the late secretory and early to mid-proliferative phases compared to the mid cycle, suggesting that sex hormones may regulate IL-8 gene expression (Arici et al., 1998a). Like IL-8, the monocyte chemoattractant protein-1 (MCP-1) mRNA levels in UFs are lower than those in the adjacent myometrium (Sozen et al., 1998). Interestingly, higher MCP-1 levels were reported in the myometrium adjacent to UFs than in the myometrium of healthy control patients (Sozen et al., 1998). In the endometrium, MCP-1 plays a key role in the control of macrophage migration in the endometrium. One study revealed that the highest levels of MCP-1 are detected when the estrogen levels are low, and MCP-1 levels are lowest around the time of ovulation, when the estrogen levels are high (Arici et al., 1999). In this context, it is possible that cytokine and chemokine expressions in the endometrium are affected by the presence of UFs, resulting in changes in the endometrial cellular function.

Several studies have demonstrated that TGF-β (Norian et al., 2009; Ciebiera et al., 2017), vascular endothelial growth factor (VEGF) (Gentry et al., 2001; Tal and Segars, 2014), platelet-derived growth factor (PDGF) (Hwu et al., 2008; Suo et al., 2009), and epidermal growth factor (EGF) (Harrison-Woolrych et al., 1994; Vollenhoven et al., 1995; Dixon et al., 2000) are differentially expressed in UFs compared to the healthy myometrium. A significantly higher expression of VEGF-A is observed in large and small UFs of younger women, indicating that angiogenesis does not depend on UFs size (Plewka et al., 2016). Estrogens upregulate PDGF expression and downregulate EGF expression in UFs (Yin et al., 2011). TGF-β and its profibrotic effects play a significant role in the pathophysiology of UFs (Ciebiera et al., 2017). At the same time, it is well known that TGF-β is involved in the initiation of menstruation and in the rapid proliferation and remodeling of endometrial tissue during the menstrual cycle and the preparation of the endometrium for implantation (Jones et al., 2006; Omwandho et al., 2010). In addition, higher levels of basic fibroblast growth factor receptor 1 (FGFR1) and basic fibroblast growth factor (bFGF) observed in women with UFs may lead to aberrant angiogenesis and HMB (Anania et al., 1997). UFs-secreted TGF-β3 provokes bone morphogenetic protein-2 (BMP2) resistance in the endometrium by downregulation of its receptor BMPR2 and leads to defective endometrial decidualization, as assessed by several decidualization markers after rhBMP2 treatment (Sinclair et al., 2011). TGF-β3 also plays a crucial role in UF-associated HMB, leading to the reduced expressions of thrombomodulin, PAI-1, and ATIII in the endometrium, likely contributing to menorrhagia (Sinclair et al., 2011). BMP7 inhibits the proliferation and decidualization in endometrial stromal cells, and it is significantly upregulated in women with abnormal menstrual bleeding (Richards et al., 2017). The higher levels of TGF-β3 observed in women with UFs inhibit the secretion of coagulation and thrombosis factors including thrombomodulin, antithrombin III, and plasminogen activator inhibitor 1 (PAI1) (Sinclair et al., 2011). Consequently, higher levels of TGF-β3 secreted by UFs dysregulate the expressions of genes associated with anticoagulant and fibrinolytic action, leading to HMB.

**FIGURE 3:**
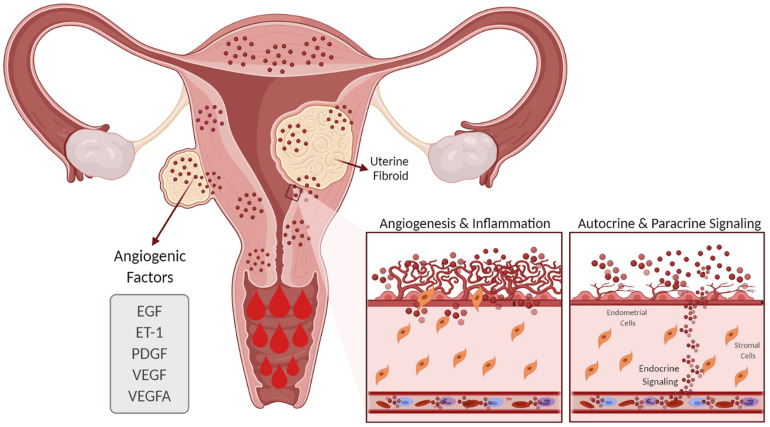
Effect of uterine fibroids (UFs) on heavy menstrual bleeding. The presence of UFs causes alterations in the endometrial vascular architecture and function, contributing to increased and prolonged menstrual bleeding. UFs influence the production of angiogenic factors such as VEGF, VEGFA, ET-1, EGF, and PDGF, among others, which support increased angiogenesis. *EGF*, epidermal growth factor; *ET-1*, endothelin 1; *PDGF*, platelet-derived growth factor; *VEGF*, vascular endothelial growth factor; *VEGFA*, vascular endothelial growth factor A.

Evidence suggests that women with UFs have increased angiogenesis and that angiogenic growth factors such as VEGF and PDGF are involved in the abnormal vasculature formation and other features of UF pathophysiology (Figure 3; Tal and Segars, 2014). Although the regulation of EGF expression in UFs compared to the myometrium is not clear, a role of EGF in UF growth is supported by the fact that the selective EGF-R blocker AG1478 inhibits UF cell proliferation (Shushan, 2004). Endometrial angiogenesis involves numerous factors and is a fundamental process for generating new capillary blood vessels during menstrual cycles and early pregnancy. It is well documented that UFs exhibit abnormal vasoconstriction including vasocongestion and dilated venous spaces (Farrer-Brown et al., 1971). A recent clinical trial of women with UFs treated with asoprisnil over the course of a year demonstrated an increase in endometrial thickness and cessation of HMB (Diamond et al., 2019). Several studies have demonstrated that angiogenic factors are differentially upregulated in UFs compared to the adjacent and distant myometrium (Anania et al., 1997). In this regard, increased expressions of angiogenic factors and their receptors in UFs may influence endometrial proliferation, ECM formation, angiogenesis, and vascularization and contribute, at least in part, to UF-associated abnormal bleeding. Taken together, changes in the number of active molecules produce an abnormal endometrial environment in UFs that leads to HMB.

#### The Impact of Uterine Fibroids on Fertility

UFs are symptomatic in approximately 30% of cases, causing HMB, pelvic pain, and infertility (Stewart, 2001; Wallach and Vlahos, 2004). The impact of UFs on fertility is complex and remains controversial. UFs are present in up to 27% of patients seeking reproductive assistance and may be the only cause of infertility in 1–3% of infertile patients (Ezzati et al., 2009; Cook et al., 2010; Guo and Segars, 2012). The most common types of UFs are intramural, submucosal, and subserosal. The clinical symptoms are influenced by UF size and anatomical location, and they are characterized by an excessive production of ECM leading to abnormal uterine contractility and decreased blood supply to the endometrium (Eldar-Geva et al., 1998; Casini et al., 2006). UFs situated completely or partially within the endometrial cavity usually cause anatomical distortion of the uterine cavity and are implicated in altering endometrial receptivity, with decreased implantation and pregnancy rates (Pritts et al., 2009).

#### Classification of UFs and Associated Endometrial Dysfunction

UFs are categorized according to their anatomical location into three main types: subserosal, intramural, and submucosa, with the most recent classification described by FIGO 2011 (Munro et al., 2011). Subserosal UFs are the least common type of UFs, protruding to the outside of the uterus (outer surface of the uterus) with minimum extension into the myometrial muscle layer. Consequently, subserosal UFs do not affect fertility, though they might cause minor alterations in uterine contractility and gamete migration. No differences in the rates of implantation, current pregnancy, and live birth were seen when comparing patients with subserosal UFs and those with no UF (Casini et al., 2006; Pritts et al., 2009).

Intramural UFs are the most common type and grow within the muscle layer. Depending on their size, intramural UFs can negatively impact fertility. There is broad agreement that intramural UFs that distort the endometrial cavity lead to decreased implantation and pregnancy rates and increased miscarriage rates. However, evidence on the effect of intramural UFs that do not distort the endometrial cavity on reproductive outcomes remains inconsistent. Most studies concur that non-cavity-distorting intramural UFs affect reproductive outcomes to a lesser degree compared to cavity-distorting intramural UFs. A recent study demonstrated abnormal Akt signaling in infertile women with non-cavity-distorting intramural UFs; these women exhibited higher expressions of Akt1, Akt2, p-Akt, and phospho-PTEN and a lower expression of PTEN mRNA in the endometrium compared to fertile women (Makker et al., 2018). In 1998, several studies demonstrated a reduction in the implantation and pregnancy rates in women with intramural UFs regardless of any cavity distortion (Eldar-Geva et al., 1998; Stovall et al., 1998). Recent studies have also shown a negative impact of intramural UFs on the implantation [16.4 *vs*. 27.7%, odds ratio (OR) = 0.62, 95%CI = 0.48–0.8] and delivery (31.2 *vs*. 40.9%, OR = 0.60, 95%CI = 0.50–0.950) rates in patients undergoing assisted reproduction when compared with patients with no UF (Benecke et al., 2005; Somigliana et al., 2007). Similarly, Pritts et al. (2009) performed a systematic review of *in vitro* fertilization (IVF) and non-IVF patients in relation to the effects of UFs on fertility. The authors confirmed the negative impact of intramural UFs on the fertility outcomes, with lower clinical pregnancy rates (OR = 0.81, 95%CI = 0.696–0.941), implantation (OR = 0.684, 95%CI = 0.587-0.796), and live birth rates (OR = 0.7, 95%CI = 0.583-0.848), along with increased spontaneous abortion rates (OR = 1.7, 95%CI = 1.226–2.489). Additional studies have reported differences in the ECM components and miRNA expression profiles in UFs with or without endometrial cavity distortion. Kim et al. (2018) reported higher expressions of estrogen receptor, matrix metalloproteinases (MMPs), and tissue inhibitors of MMPs (TIMPs) and lower expressions of miR-29c and miR200c in UFs with compared to UFs without distortion of the endometrial cavity.

**FIGURE 4:**
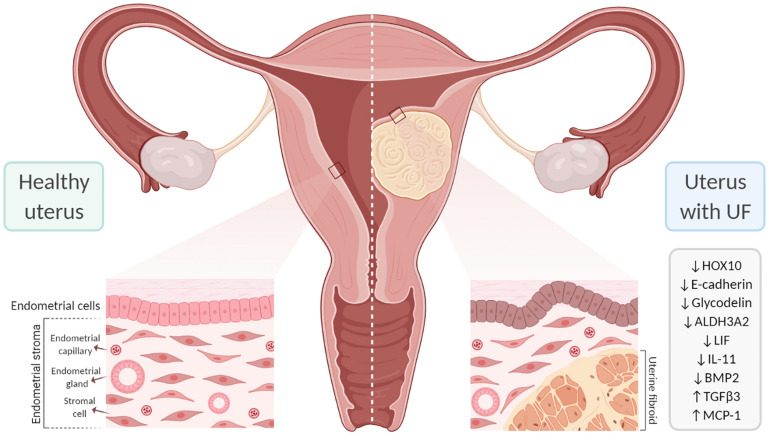
Effect of uterine fibroids (UFs) on endometrial receptivity and implantation. The presence of UFs impacts endometrial gene expression, contributing to failure in endometrial receptivity. In addition, submucosal UFs can distort the uterine cavity, which interferes with embryo implantation and placentation, likely affecting fertility. *ALDH3A2*, aldehyde dehydrogenase 3 family member 2; *BMP2*, bone morphogenetic protein 2; *HOX10*, homeobox gene 10; *IL-11*, interleukin 11; *LIF*, leukemia inhibitory factor; *MCP-1*, monocyte chemoattractant protein-1; *TGF-*β*3*, transforming growth factor beta 3.

Submucosal UFs generally bulge into the uterine cavity and are more likely to affect fertility due to their proximity to the endometrium, distortion of the endometrial cavity, and interference with embryo implantation and placentation (Figure 4). The harmful influence of submucosal and large cavity-distorting UFs on reproductive outcomes is well recognized and guides clinical management (Pritts et al., 2009; Olive and Pritts, 2010). Approximately 26% of women have had submucosal UFs by the time they reach their late 40s (Baird et al., 2003). In their meta-analysis, Pritts et al. (2009) revealed that patients with submucosal UFs have reduced clinical pregnancy rates [relative risk (RR) = 0.363, 95%CI = 0.179–0.737], implantation rates (RR = 0.318, 95%CI = 0.123–0.649), and ongoing pregnancy/live birth rates (RR = 0.318, 95%CI = 0.119–0.850) and an increased risk of spontaneous miscarriage (RR = 1.678, 95%CI = 1.373–2.051). Interestingly, a recent retrospective study analyzed the long-term fertility consequences after myomectomy relative to the number of UFs removed. They found a direct relationship between the number of UFs removed and fertility problems. UF patients with more than six UFs removed were less likely to achieve pregnancy or carry a birth to full term, and more likely to need fertility treatment, compared to women with six or fewer UFs removed (Shue et al., 2018). Infertility is a multifaceted disorder, and the precise influence of UFs on pregnancy outcomes is difficult to assess. However, it is well documented that submucosal and intramural UFs that alter the uterine cavity have a negative impact on endometrial receptivity, implantation, and live birth rates (Bulletti et al., 2004; Casini et al., 2006).

#### Effect of Uterine Fibroids on Endometrial Receptivity and Implantation

Implantation is a process that involves a highly regulated and synchronous development of the embryo and the endometrium to make it amenable to implantation, a process that occurs between 7 and 10 days after ovulation and is known as the window of implantation (WOI) (Achache and Revel, 2006). Endometrial receptivity allows for implantation of the embryo, and it is a multidimensional process of molecular events influenced by hormones, cytokines, growth factors, and other signaling molecules. Any abnormality can lead to implantation failure, early pregnancy loss, or problems conceiving.

#### Critical Factors in Endometrial Implantation

The family of homeobox genes comprises 39 HOX transcription factors that are fundamental to the proper development of the female reproductive tract and to endometrial development during the menstrual cycle (Du and Taylor, 2015). HOX genes are also crucial to endometrial receptivity; the most relevant are *HOXA10* and *HOXA11*, which are expressed in the endometrium throughout the proliferative phase and reach a peak in the mid-secretory phase under the influence of progesterone (Taylor et al., 1998, 1999). The proteins encoded by these genes affect endometrial receptivity by inhibiting or activating target genes including β*3-integrin* and *Emx2* (Figure 3; Du and Taylor, 2015). *HOXA10* and *HOXA11* are downregulated in the secretory phase of women with low rates of implantation (Taylor et al., 1999; Bagot et al., 2000). *HOXA10* expression is also reduced in the endometrium of women with submucosal UFs; the reduction is detected throughout the endometrium, but is significantly reduced in the endometrium covering submucosal UFs (Rackow and Taylor, 2010). Endometrial expressions of *HOXA10* and *HOXA11* increase after myomectomy of intramural UFs, but not submucosal UFs (Unlu et al., 2016). A study analyzing endometrial *HOXA10* and *HOXA11* levels during the WOI in infertile women with intramural UFs found significantly decreased levels of *HOXA10* and *HOXA11* and a slight decrease in E-cadherin compared to healthy fertile women (Makker et al., 2017). HOX genes are regulated by BMP2; consequently, increased endometrial resistance to BMP2 could contribute to the low *HOXA10* and *HOXA11* levels in the endometrium of women with UFs (Sinclair et al., 2011; Doherty and Taylor, 2015). Endometrial *HOXA10* and *HOXA11* expressions are upregulated by progesterone and 17β-estradiol during the mid-secretory phase and improve endometrial receptivity (Cermik et al., 2001).

Numerous studies have analyzed gene expression in endometrial tissue from women with intramural and/or submucosal UFs and women without UF to investigate correlations with endometrial receptivity. These studies identified several genes that are differentially expressed during the mid-secretory phase. However, the genes reported to be significantly altered vary across studies, and the precise mechanism by which gene alterations affect receptivity remains unclear (Gómez et al., 2015; Aghajanova et al., 2017). One study investigated gene expressions during the WOI in women with and without intramural UFs and in those without and with observed alterations in aldehyde dehydrogenase 3 family member 2 (ALDH3A2) and the glycodelin expression in women with intramural UFs of >5 cm (Horcajadas et al., 2008). The findings indicated that larger intramural UFs may have a more significant impact on endometrial gene expressions; more studies are needed to better understand this association. In addition, analyses of gene expressions during WOI revealed endometrial dysregulation of the molecules involved in cell adhesion. One study reported the downregulation of E-cadherin and the increased expressions of integrin and osteopontin in women with non-cavity-distorting intramural UFs, as well as an increased pinopode formation (Bentin-Ley, 2000; Apparao et al., 2001; Lessey, 2002). Recently, a very well-designed study investigated the association between the expressions of endometrial receptivity genes and essential endometrial functions such as decidualization, proliferation, and apoptosis in women with UFs. Women with UFs demonstrated significantly altered transcriptional patterns throughout the menstrual cycle compared to healthy women, although no significant differences were observed in the expressions of receptivity and decidualization genes (Aghajanova et al., 2017).

A significant number of endometrial events are crucial to boost endometrial receptivity, which requires a complex interchange among paracrine and autocrine factors such as cytokines, chemokines, their receptors, and secondary messengers. The surge in progesterone following ovulation leads to decidualization of the endometrium and is characterized by rising levels of VEGF, prostaglandins, and immune cells (macrophages and natural killer cells) (Wang et al., 2000; Lee et al., 2015). During decidualization, there is an increase in endometrial blood vessel permeability and the production of cytokines necessary for implantation, such as leukocyte inhibitory factor (LIF), which is a marker of the WOI. Successful embryo implantation is the result of a bidirectional invasive process that is coordinated by decidual markers including LIF, prolactin, insulin-like growth factor-binding protein 1 (ILGFBP1), and IL-11. LIF and IL-11 are crucial decidual markers for embryo implantation (Stewart et al., 1992; Dimitriadis et al., 2005; Hasegawa et al., 2012). These factors bind to their respective ligand-specific receptors, LIFR and IL-11R, which share the same signal transduction target, gp130. Murine studies have demonstrated that the gp130 pathway is vital for embryo implantation and that its inactivation leads to failure of implantation (Ernst et al., 2001). LIF is a key player in the endometrium and is required for decidualization; embryos from mice lacking LIF are unable to implant in the endometrium of mice lacking LIF, but are able to implant in the endometrium of wild-type mice (Stewart et al., 1992; Robb et al., 1998). In several human studies, the LIF levels were shown to be upregulated in the luteal phase and reach their highest expression levels during WOI; in contrast, women with submucosal UFs show decreased levels of LIF during the luteal phase. Additionally, a recent study demonstrated that LIF is significantly downregulated in the endometrium of women with large (≥3 cm) and non-cavity-distorting intramural UFs (Pier et al., 2020). Once the embryo has attached to the endometrium, IL-11 moderates trophoblast invasion. Reduced levels of IL-11 lead to decreased levels of natural killer (NK) cells in the secretory endometrium and to early pregnancy loss in mice and humans (Hasegawa et al., 2012). The presence of submucosal UFs leading to reduced IL-11 during the WOI may thus cause implantation problems (Hambartsoumian, 1998).

Progesterone is vital for decidualization and the production of immune cells, such as macrophages and NK cells. Macrophages secrete crucial cytokines for implantation, such as LIF, and they are critical during trophoblast invasion and placental development (Miura et al., 2006; Jensen et al., 2012; Helige et al., 2014). During the WOI, NK cells are the predominant immune cells and are critical regulators of immunotolerance, trophoblast migration and invasion, and angiogenesis. NK cells secrete VEGF and placental growth factor, both of which play a role in trophoblast invasion and maternal uterine vasculature remodeling (Wang et al., 2000; Tayade et al., 2007). Murine studies have shown that mice lacking NK cells are able to achieve pregnancy, but they have significant rates of fetal loss, preeclampsia, and intrauterine growth restriction (King, 2000). Human studies of the mid-secretory endometrium of women with and without UFs demonstrated a rise in macrophage production and a reduction in the production of NK cells (Kitaya and Yasuo, 2010b). The dysregulated levels of NK cells and macrophages lead to abnormal endometrial function and may contribute to failure in endometrial receptivity and implantation. Moreover, women with UFs have greater expression of MCP-1, which is associated with a higher density of macrophages and PGF2α and an inflammatory effect in the endometrium. Recent studies analyzed the endometrial flushing levels to check for endometrial receptivity markers and found a significant reduction in the IL-2 levels, but no significant differences in PGF*2*α, ανβ*3* integrin, and TNF-α in women with UFs compared to healthy women (Demir et al., 2017, 2019). The same study found only a slight increase in glycodelin in women with UFs.

Growth factors play crucial roles in decidualization and implantation, and they are dependent on progesterone. Important growth factors include members of the TGF-β family, such as heparin-binding epidermal growth factor (HB-EGF), which stimulates the secretion of BMP2 and its downstream target member WNT4 (Paria et al., 2001; Li et al., 2013). The stimulation of BMP2 levels by progesterone seems to be essential for WNT4 activation and, consequently, implantation. Murine studies have demonstrated that mice lacking BMP2 are incapable of achieving endometrial decidual differentiation (Lee et al., 2007; Li et al., 2007). Though embryo attachment is achievable, the lack of decidual differentiation leads to faulty implantation and pregnancy loss. Human studies have shown that BMP2 resistance occurs in submucosal UFs, and this resistance adversely affects cell proliferation and differentiation, leading to impaired decidualization and faulty implantation. Most women with submucosal UFs secrete higher levels of TGF-β3, a factor that impairs the signaling of BMP2 in the endometrium and is associated with defective embryo implantation in UFs (Lee et al., 2007). In general, studies have shown a direct correlation of the expression level of TGF-β and UF burden. Significantly, lower levels of BMP2 are associated with decreased endometrial stromal cell expressions of *HOXA10* and LIF (Sinclair et al., 2011), a higher rate of spontaneous abortions, and a lower rate of implantation.

## Discussion

In summary, it is critical to understand how UFs affect the normal physiology of the endometrium and lead to two of the most common endometrial dysfunctions: HMB and subfertility. There is a crucial need for non-invasive treatment options and anti-fibroid therapeutics, which disproportionally affect African American women and cause a significant burden on women’s everyday quality of life. In general, it is believed that UFs cause abnormal menstrual bleeding by altering local and distant endometrial gene expressions, which subsequently alters endometrial function. UFs affect the normal endometrium by modifying the vascular architecture, impairing the normal contractility, and altering the production of angiogenic factors (Figure 2; VEGF, VEGFA, and ET-1), cytokines (TNF-α), chemokines, growth factors (TGF-β, bFGF, EGF, PDGF, and PDEF), prostaglandins (PGF2α), and factors involved in coagulation and fibrinolysis (PAI1, tPA, ATIII, and TM). It is of paramount importance to investigate the mechanisms underlying HMB and subfertility secondary to decreased receptivity and implantation in women with UFs and to better understand the processes underlying UF pathophysiology so that new therapeutics can be identified.

Overall, some of the main factors that affect fertility in women with UFs are distortion of the endometrium and uterine cavity, interference with the normal patterns of endocrine function, abnormal uterine vascularization, endometrial inflammation, and dysfunctional uterine contractility (Rogers et al., 2008; Rackow and Taylor, 2010; Norian et al., 2012). The impact of submucosal and intramural UFs that distort the uterine cavity is well documented, with negative effects on endometrial receptivity, implantation, and pregnancy, increased miscarriage rates, and decreased live birth rates (Pritts et al., 2009). However, the effect of endometrial non-cavity-distorting intramural UFs remains inconsistent, with most studies concurring that they affect reproductive outcomes to some extent, but to a lesser degree. Several mechanisms have been proposed to explain the effects of UFs on fertility, including simple physical impedance by obstructing the transport of gametes or embryos. Other mechanisms delay implantation by altering the normal pattern of myometrial contractions (Lyons et al., 1991), inducing a chronic inflammatory reaction and fibrosis, and impairing endometrial decidualization in the mid-luteal WOI by significantly reducing the concentrations of both macrophages and uNK cells in the EMJ zone (Kitaya and Yasuo, 2010a; Kido et al., 2014). Physical disruptions of the EMJ and alteration of the steroid receptors, acceleration of myometrial peristalsis in the mid-luteal period, and upregulation of the prolactin and aromatase levels are additional mechanisms by which UFs may affect fertility (Brosens et al., 2003; Tocci et al., 2008; Yoshino et al., 2010).

**FIGURE 5:**
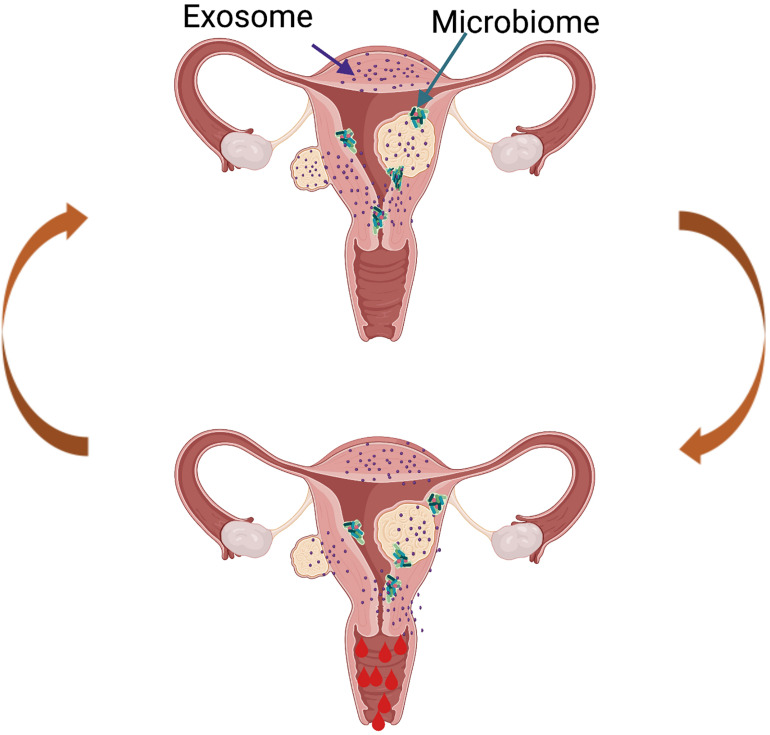
Proposed model of future directions in the uterine fibroid (UF) field. The presence of UFs causes alterations in the healthy endometrial microbiome and changes in the exosome content, leading to increased factors involved in cell proliferation, angiogenesis, and inflammation, among others. This creates a vicious cycle contributing to increased and prolonged heavy menstrual bleeding in women with UFs.

The field continues to advance with innovative studies examining the impact of UF-derived exosomes on the human endometrium and the impact of UFs on the endometrial microbiome (Figure 5), and this is an area of active investigation in our lab. Though limited data are available, it is hypothesized that the UF secretome is delivered to the human endometrium *via* exosomes, which affect critical biological functions including menstrual bleeding, endometrial receptivity, and implantation. Exosomes are vesicles ranging from 30 to 150 nm, derived from the fusion of multivesicular bodies with the plasma membrane and are secreted by a variety of cells. They consist of a lipid bilayer membrane and contain various functionally active proteins, mRNAs, and miRNAs that are delivered to target cells and tissues. Exosomes play crucial physiological roles as mediators of intercellular cell signaling between neighboring cells and distant tissues, acting independently but synergistically with soluble factors and hormones (Di Pietro, 2016). One preliminary study by Brakta et al. (2015) characterized exosomes derived from human UF stem cells. The authors described a significantly higher exosome production and an increased cell proliferation under hypoxic conditions compared to normoxic conditions. There is consensus that the vagina is colonized with bacteria, while the uterus and the rest of the upper reproductive tract are considered sterile. However, recent studies have demonstrated the presence of bacteria continuum throughout the reproductive female tract, challenging the traditional dogma (Chen et al., 2017). The role of the endometrial microbiome under normal physiological conditions and in disease conditions is an area of active investigation, and currently, research in our team is focusing on the impact of an altered microbiome in women with UFs. Most endometrial microbiome studies have focused on pregnancy, leaving a significant gap in our understanding of the role of the microbiome in UFs. The term “estrobolome” was recently coined to describe the secretion of circulating estrogens and their impact on the microbiome. We predict that the estrobolome plays a crucial role in UF pathophysiology, as fibroids are estrogen- and progesterone-dependent, and endometrial microbiome dysbiosis may contribute to modifications in the normal actions of estrogen in women with UFs. In the absence of a well-defined catalog of endometrial microbiota, we postulate that the presence of UFs impacts the composition of the endometrial microbiome (Figure 5). Recently, Dr. El Andaloussi was the first to report a 16S rRNA screen of the microbiome in human UF. His study demonstrated a higher alpha and beta diversity in the endometrium of women with UFs compared to a healthy endometrium (El Andaloussi et al., 2020). Investigating the effect of UFs on the endometrial microbiome may lead to the development of novel non-hormonal, non-invasive treatment options for UFs and its associated endometrial dysfunctions. Overall, there is an urgent need to discover novel therapeutics for the treatment of UFs, a common disease with a huge personal and societal burden globally and in the United States, affecting critical reproductive functions like menstrual bleeding and fertility.

## Author Contributions

AN and MVB designed the review, performed the literature search, and wrote the manuscript. QY and AA-H performed revisions and critically discussed and reviewed the complete manuscript. All authors contributed to the article and approved the submitted version.

## Conflict of Interest

The authors declare that the research was conducted in the absence of any commercial or financial relationships that could be construed as a potential conflict of interest.
